# Triadic relationships in home care nursing: an integrative review of the views and experiences of older couples and nurses

**DOI:** 10.1186/s12912-025-03378-1

**Published:** 2025-06-27

**Authors:** Katharina Niedling, Stefanie Richter, Kerstin Hämel

**Affiliations:** 1 School of Public Health, Bielefeld University, Universitätsstraße 25, 33615 Bielefeld, Germany; 2 Faculty of Social and Health Care Sciences, OTH University of Applied Sciences Regensburg, Seybothstraße 2, 93053 Regensburg, Germany; 3 Department of Nursing Science, Faculty of Social Sciences, University of Vienna, Alser Strasse 23/12, Vienna, 1080 Austria

**Keywords:** Chronic illness, Marital dyad, Older couples, Qualitative research, Long-term care, Spouses, Home nursing, Home care services, Caregivers, Relationship-centred care

## Abstract

**Background:**

The need for long-term home care for older people with chronic health conditions is rising, and family members are bearing the brunt of this care work. Caring for a partner is a common scenario. Caregiving partners are often of advanced age themselves and have limiting health conditions of their own. Nurses need to consider the specific characteristics of partner caregiving to provide relationship-centred care. This review aims to analyse perceptions of the triadic relationship between older persons with long-term care needs, their caregiving partners and nurses and to identify approaches that can be supportive approaches that can help couples to cope with at-home care situations.

**Methods:**

This integrative review following the method outlined by Whittemore and Knafl focuses on qualitative and mixed methods studies. A systematic search in the PubMed and CINAHL databases served to identify studies dealing with experiences and perceptions of triadic care arrangements in home-based care from the perspectives of nurses, care recipients and caregiving partners. Eight studies met the inclusion criteria and investigated perceptions of the triadic relationship between nurses providing home care and older couples. They were analysed using thematic analysis.

**Results:**

The results show that triadic relationships in home-based care are shaped by social dynamics, role conflicts and barriers to communication. Although many couples are initially reluctant to integrate nursing professionals into home caregiving, mutual trust can develop when personnel continuity is given and sustained relationship work is performed. Clear communication, clarity on the distribution of tasks and proactive knowledge sharing by nurses facilitate cooperation. Caregiving partners desire support with self-care to preserve their ability to continue in their caregiving roles in the long term.

**Conclusions:**

Understanding the dynamics of triadic relationships in nursing care and the care situation in couple relationships enables nurses to provide more targeted support to older couples. Proactive communication, role clarity and continuity of care can reduce the burden shouldered by caregiving partners and improve care quality. Further research focusing on strategies for strengthening relationship-centred interventions in home-based care is desirable.

**Clinical trial number:**

Not applicable.

**Supplementary Information:**

The online version contains supplementary material available at 10.1186/s12912-025-03378-1.

## Background

In view of increasing global demand for care for older people with chronic conditions and functional limitations, long-term care is an important field of action in nursing [1, 2]. Ageing in place has become a major objective of long-term care policies in many countries [3, 4]. High-quality home care services are a cornerstone for realising this ambition. At the same time informal caregivers shoulder the main burden of care in the home and this places many of them under severe psychological, physical and financial strain [5, 6]. In this light, it is important that nursing professionals in home-based care address both the needs of care recipients and informal caregivers [7, 8]. To support self-management and care in the home, nurses need to understand the lifeworlds and relationships of care recipients and informal carers and the effects on them of routines changing due to chronic illness and care dependency.

Caring for a spouse or a partner in the home is a common caregiving scenario [5]. In many cases, partners have lived together for years and established regular activities and stable roles. Progressive, chronic conditions and a partner becoming care-dependent make everyday life challenging and disrupt the familiar patterns of the couple relationship [9]. Both the caregiving partner and the person with chronic illness can experience existential insecurity and “biographical shocks and crises” [10].

Caregiving within couples is less supported by services than other caregiving models such as adult children caring for parents [11, 12]. Couples are often particularly hesitant to seek or accept professional support, which leads to late uptake of services – often only when care needs or caregiver burden become unmanageable due to an acute health issue [13, 14]. Couples in a caregiving relationship are at risk of becoming socially isolated [15]. It follows that reaching this target group more effectively and developing suitable support options is an important task for nurses [9, 16]. In this context, we understand care as a triadic relationship between the nurse, the care recipient and the caregiving partner.

### A theoretical and conceptual discussion of triadic relationships in nursing care situations

The model of relationship-centred care (RCC) will be drawn on here to gain a better understanding of the opportunities and challenges that triadic relationships in home-based care involving older couples present. According to Nolan et al. [17], RCC opens up the perspective that care takes place in relationships. The nature and quality of relationships are therefore of central importance for healthcare delivery. The interactions between self-care, informal care and professional care are fundamental to successful care situations. For example, mutual respect between nurses and informal caregivers is desirable to foster reciprocal, complementary and symmetrical relationships and facilitate self-determination for persons with long-term care needs [17].

In the dyadic structure of a couple in a caregiving relationship, the symmetry of the couple relationship is in jeopardy. For many caregiving partners, the nursing care relationship increasingly moves into the foreground and relegates the couple relationship into the background [15, 18]. The care situation threatens the quality of the relationship, and this in turn can negatively affect the health of both partners [19–21]. This means that nurses need to focus attention also on the relationship between the partners [9, 17].

RCC is based on four related principles: “(1) that relationships in health care ought to include the personhood of the participants, (2) that affect and emotion are important components of these relationships, (3) that all health care relationships occur in the context of reciprocal influence, and (4) that the formation and maintenance of genuine relationships in health care is morally valuable” [22] (p.3).

Nolan et al. [23] expanded the relationship-centred care approach by developing the Senses Framework, which promotes cooperation between care recipients, informal caregivers and professionals. It highlights six key dimensions – security, continuity, belonging, purpose, achievement and meaning – that should guide professionals towards enhancing care quality. As Ryan [24] emphasizes, considering the perspectives of both care recipients and informal caregivers is essential to relationship-centred care. A well-coordinated collaboration between informal caregivers and nurses positively impacts the well-being of all parties involved [10].

### Objectives and research question

This review focusses on triadic care scenarios in home-based long-term care involving nurses and older couples. It focuses on a typical family caregiving scenario that has, nevertheless, received little attention up to now in nursing research. It aims to analyse the perceptions of those involved in these care triads and to identify ways in which nurses^1^ can support partner caregiving in the home. Our research questions are:

- How is collaboration within the care tried experienced by care recipients, caregiving partners, and nurses?
- How do those involved – care recipients, caregiving partners and nurses – view the support approaches adopted by nurses to the at-home care situations of couples?
- What are the facilitators of and barriers to collaboration within the care triad?

## Method

### Study design

We conducted an integrative review based on the method of Whittemore and Knafl [25] to investigate the research questions. Our approach, which we described in detail below, considered the PRISMA 2020 reporting guidelines for systematic reviews and meta-analyses [26]. This approach was chosen as it enabled a comprehensive and flexible synthesis of diverse sources and study designs. It was particularly well-suited for addressing exploratory research questions in a relatively under-researched field, such as triadic care relationships involving older couples and nurses [26]. To enhance methodological transparency and reproducibility, several elements of systematic review methodology were incorporated. These included a structured search strategy across multiple databases, the use of predefined inclusion and exclusion criteria, and the presentation of the study selection process using a PRISMA flow diagram. While integrative reviews are methodologically more flexible than systematic reviews, adopting these components allowed for increased clarity and scientific rigour in reporting.

### Search strategy

A search for studies was conducted in the PubMed (via MedLINE) and CINAHL databases. The search strategy consisted of keywords and synonyms in the thematic areas of *home care*, *couple (relationship)*,* old persons*,* nurses* (see Table 1). A detailed overview of the search strategy and the search strings used can be found in Table 1.

In addition, the bibliographies of all included studies were checked to see whether they contained studies that met the inclusion criteria.

**Table 1:** Terms used in search strategies

Thematic area	MeSH terms*	Relevant key words**
Home Care	Home Care Services	Home Care Services, Domiciliary Care, Home Health Nurse, Home Health Care, Home Healthcare, Home Nurse, Home Care, Care at Home, Family Care, Relative
Couple (relationship)	Marriage Spouses	Marriage, Spouse, Couple, Partner, Wife, Wive, Husband,
Old Persons	Aged	Aged, Old Person, Elder
Nurses	Nursing Nursing Care Family Nursing Community Health Nursing Public Health Nursing	Nursing Nursing Care Family Nursing Community Nurse Public Health Nursing

*MeSH terms were combined using the Boolean operator AND, and the search terms within each box were combined with OR

**Keywords were searched using truncation and phrase symbols when appropriate

### Inclusion and exclusion criteria

Qualitative and mixed methods studies focusing on the subjective experience of the home-based care situation of older (often married) couples were included. They looked at older couples who lived together and availed of support from nurses. The focus was on older couples who were 60 years of age or older, which was a requirement for inclusion in this review. We looked for studies that presented subjective views and experiences of home-based care situations from the perspective of at least one of the three roles in a triadic nursing relationship. Quantitative studies were excluded, as they did not enable a comprehensive picture of subjective views and experiences to emerge. Clinical reports, care assessment studies and contributions describing the development, delivery or evaluation of interventions were excluded so that only exploratory, descriptive and/or theory-generating studies on home-based care situations remained. Studies that did not foreground home-based care – and looked, for instance, at short-term respite care or long-term inpatient care – were excluded. Studies on technology-intensive home care, such as home ventilated patients, were also excluded, as this is a highly specialised field of care.

The search was limited to studies in English and German that were published in the timespan between 1 January 1991 and 28 June 2024.^2^ Studies from the 1990s onwards were included because models such as relationship-centred care came into focus in healthcare research and other scholarly research around the turn of the millennium.

The reference management software Citavi was used to support the initial identification of duplicates. A screening of titles and abstracts was carried out before a full-text screening was performed. The screening steps were carried out by the first author, a co-author (a different one in each case) and/or a fourth, external person. The screeners worked independently and any disagreement on inclusion or exclusion was resolved by the co-authors discussing their assessments until a consensus was reached.

### Data analysis

A tabular overview of the studies included in the review was prepared. The results of all the contributions were summarised by the first author as recommended by Whittemore and Knafl [25]. A one-pager [27] was prepared for each study that excerpted its content with regard to the three research questions of relevance for the review:

- How is collaboration within the care tried experienced by care recipients, caregiving partners, and nurses?
- How do those involved – care recipients, caregiving partners and nurses – view the support approaches adopted by nurses to the at-home care situations of couples?
- What are the facilitators of and barriers to collaboration within the care triad?

The perspectives of all three parties (caregiving partner, care recipient, nurse) were carefully reproduced as explicitly as possible in these summaries. Structured one-pagers were used to systematically summarise each included study, capturing key content, underlying concepts, theories, and methods. This approach helped ensure consistency across sources and provided a clear foundation for comparison and thematic analysis. It was acknowledged that summarizing the studies prior to analysis might have introduced interpretive bias – particularly in the context of a research perspective shaped by the German care system, which is oriented towards task-based delivery. To mitigate this, the one-pagers were revised and discussed multiple times, and thematic analysis was conducted directly from the original results sections. Comments on the summaries, with reference to the study aims, were provided by both co-authors. After several rounds of revision, the summaries were considered clear and were found to reflect the viewpoints of the involved parties on the issues under investigation. The thematic analysis of the summaries then followed the six-phase process outlined by Braun & Clarke [28]: familiarization with the data, generating initial codes, searching for themes, reviewing themes, defining and naming themes and producing the report. The process was inductive and data-driven, with no predefined coding framework. The initial coding was conducted by the first author. Coding decisions and emerging codes were then reviewed and discussed by all three authors to ensure consistency and shared understanding. All authors engaged in repeated discussions to refine and consolidate the emerging themes. Reflexivity was maintained throughout, acknowledging our backgrounds within a task-oriented German-speaking care context and its potential influence on data interpretation.

For this purpose, the excerpts on the three questions (the main categories) were each coded inductively by identifying recurring aspects. The team of authors merged codes/aspects several times and this condensation process solidified the robustness of the descriptions and led to the identification of commonalities between the contributions. The category system is shown in the results section (Table 4).

## Results

### Search results

After removing duplicates, 896 studies were retrieved. 421 studies were excluded after an initial screening of titles and abstracts. Studies were excluded because of their design (quantitative studies, reports on interventions or evaluations) or because they related to a setting other than long-term care in the recipient’s home (e.g. short-term respite care, nursing home or hospital care).

475 studies were included in an in-depth abstract screening. 403 of these studies were subsequently excluded because they did not deal with a nursing professional providing home-based care or with couples.

**Fig. 1:**
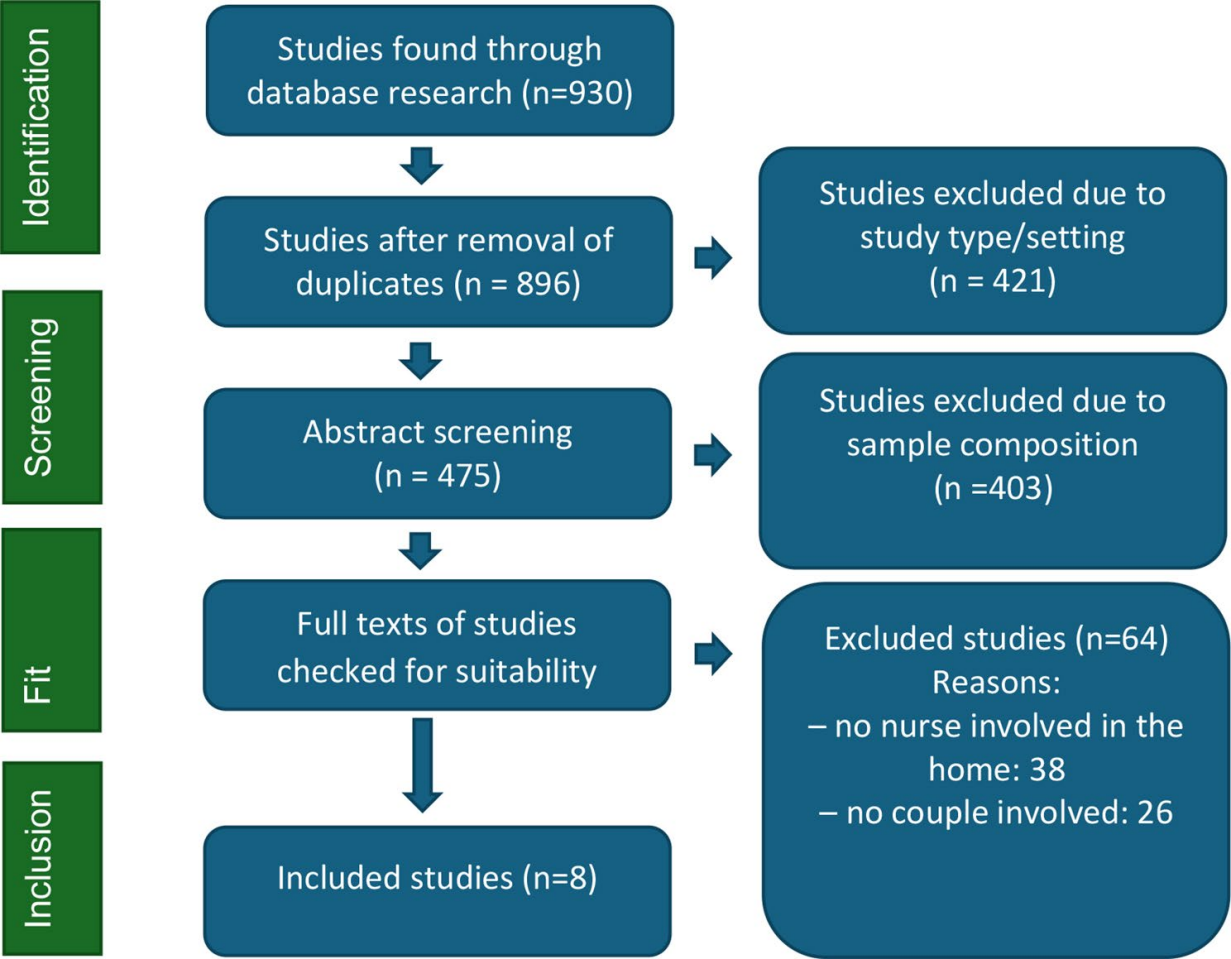
PRISMA flow chart of search results

Full texts of 72 studies were checked for suitability by the first author. Studies that did not clearly meet the criteria for inclusion or exclusion were discussed by the team of authors until a consensus was reached. This screening resulted in 64 exclusions. The reasons for the exclusions were listed in Fig. 1. The 8 studies that remained were analysed to answer our research questions.

### Quality assessment

The Critical Appraisal Skills Programme (CASP), CASP Checklist for Qualitative Studies [29] and Systematic Reviews [30] were used for the quality assessment. This showed that the eight studies had “no or minor” (*n* = 3), “minor to moderate” (*n* = 2), “moderate” (*n* = 2) and “moderate to severe” (*n* = 1) restrictions.

### Overview of the included studies

The 8 qualitative empirical studies included were conducted in the USA (*n* = 3), Germany (*n* = 2), Canada (*n* = 1), Iceland (*n* = 1) and Sweden (*n* = 1). Most of the studies (*n* = 5) focused on the perspectives of caregiving partners. Three (3) studies analysed the perspective of all three members of the care triad (caregiving partner, care recipient, nurse).^3^ Table 2 provides an overview of the studies that were included (Table 3).

**Table 2:** Quality appraisal based on CASP, critical appraisal skills programme (2018)

No.	Contribution	Was there a clear statement of the aims of the research?	Is a qualitative methodology appropriate?	Was the research design appropriate to address the aims of the research?	Was the recruitment strategy appropriate to the aims of the research?	Was the data collected in a way that addressed the research issue?	Has the relationship between researcher & participants been adequately considered?	Have ethical issues been taken into consideration?	Was the data analysis sufficiently rigorous?	Is there a clear statement of findings?	Overall assessment of limitations
1	Bauernschmidt et al. (2014)	+	+	+	+	+	0	+	+	+	Minor to moderate
2	Hash (2006)	+	+	0	+	+	0	0	0	+	Moderate to severe
3	Hudson, Janella; at al. (2019)	+	+	+	0	+	0	+	+	+	Moderate
4	Jonsdottir et al. (2011)	+	+	+	+	+	+	+	0	+	Minor to moderate
5	Lethin et al. (2016)	+	+	+	+	+	+	+	+	+	Minor
6	Lindgren et al. (1993)	+	+	+	+	+	0	0	+	+	Moderate
7	Münch (2020)	+	+	+	+	+	+	+	+	+	Minor
8	Ward-Griffin et al. (2000)	+	+	+	+	+	+	+	+	+	Minor

+ = Yes; - = No; 0 = Can´t tell

**Table 3:** Summary of key characteristics of the included studies

No.	Author(s)	Country	Aim	Study design (method of data collection and analysis)	Sample (study participants, age, duration of partnership, duration of care)	Perspective of A Caregiver B Cared-for C Nurse	Main results concerning the review questions
1	Bauernschmidt et al. (2014)	Germany	To describe the experiences of husbands in caring for women with dementia and implications for the design of home care arrangements	Narrative interviews; phenomenological- interpretative analysis	*N* = 10 caregiving husbands Age: 67–95 years Duration of marriage: 22–67 years Duration of care: 1.5–30 years	A	The caregiving husbands try to organize the care of their wives according to their individual needs and to maintain the everyday routines they have established as a couple. They see it as necessary that nurses’ support is oriented towards their lifeworld. To be able to hand over caregiving tasks, the husbands need trust in the nurses. This is established through consistency (e.g. by always having the same nurses in charge). Husbands are more likely to accept help if they are not isolated but also integrated into social contexts beyond the couple relationship.
2	Hash (2006)	USA	To investigate the caregiving experiences of same-sex partners in middle and old age and/or their experiences after relocation or the death of the cared-for partner	Semi-structured interviews; constant comparison method	*N* = 19 caregiving partners (9 female, 10 male) Age: 50–77 years Duration of partnership: 2–34 years Duration of care: 4 months to 22 years	A	The caregivers describe that some practices of organisations and professionals were insensitive towards same-sex partners and partner were often not regarded as next of kin. Respondents describe that supportive professionals support the couple relationship and refer respondents to other supportive professionals and services.
3	Hudson, Janella et al. (2019)	USA	To describe the communication between patients with a cancer diagnosis, caregiving spouses, and nurses during home hospice visits focusing on phases of role changes and conflict	Secondary, qualitative analysis of dyad interactions; constant comparison method	*N* = 19 caregiver/cared-for dyads (3 male, 16 female caregivers; 16 male, 3 female cared-for); *N* = 19 home hospice nurses (19 female) Age: Caregiving partners: 50–83 years Cared-for: 53–87 years Duration of partnership: 2–65 years (⌀ 35.9 years) Duration of care: not specified	A, B, C	As a mediator, the hospice nurse helps to convey information and feelings that may be too difficult for caregivers and patients to communicate directly. The nurse helps to facilitate an understanding of tensions and serves as a safe channel for the spouses to share these feelings with each other.
4	Jonsdottir et al. (2011)	Iceland	To describe the experience of an evolving partnership between chronically ill patients with breathing difficulties, their families and home care nurses.	Narrative interviews with patient and/or close family member; narrative analysis	*N* = 8 cared-for (5 female, 3 male); *N* = 7 caregivers (5 spouses) Age: Caregivers: not specified Cared-for: 55–70 years Duration of partnership (only couples): not specified Duration of care: not specified	A, B, C	All participants focus on looking at the health situation as a whole, seeing health problems in context, and promoting not only the health of the patient but also that of the family. Nurses and families share information on family conventions, frictions, desires, and opportunities. Knowledge sharing creates an understanding of how to manage the disease. The sense of safety proves to be the most important component for the families in the nurse-patient-family relationship. Feeling secure helps them to cope with the illness and leads to greater stability.
5	Lethin et al. (2016)	Sweden	To investigate family caregivers’ experiences of formal care when caring for a person with dementia, through the stages of the disease.	Focus groups; method of analysis not described	*N* = 23 caregivers (13 spouses, 10 children) (13 female, 10 male) Age: Caregivers: 45–88 years Cared-for: 68–98 years Duration of partnership (only couples): not specified Duration of care: not specified	A	The caregiving partners would like to be prepared for the caring role by nurses through shared knowledge and structured information about care and support options and the specific illness. Caregivers describe nurses as reactive (rather than proactive) to caregivers’ needs. They want to work closely with nurses to continue home care, support daily life, and maintain independence. This requires support that is tailored to individual needs and corresponds to the relevant stage of the person’s illness. However, they often feel alone and isolated in their new role as caregivers because nurses do not provide the expected support.
6	Lindgren et al. (1993)	USA	To understand the situation of spouse caregivers and how their needs may be perceived and understood by professional healthcare providers	Open-ended interviews; analysis with data categorization	*N* = 10 spouse caregivers (6 females, 4 males) Age: 59–77 years Duration of marriage: 14–39 years Duration of care: 1–9 years	A	Caregivers are looking for individual support that fits their needs. They want nurses to teach them skills so that they can provide care. In addition to instrumental support, they especially want information about the course of the disease, emotional/social support and comfort. The feedback of the nurses is important to the caregivers because this information gives the caregiving partners a feeling of security. In this sense, nurses act as consultants and teachers.
7	Münch (2020)	Germany	To describe the subjective experiences of caregiving partners and the sociospatial challenges within the specific care situation of people with dementia being cared for at home	Problem-centred interviews; Situational analysis	*N* = 16 caregivers (8 male, 8 female) Age: 64–95 years Duration of marriage: not specified Duration of care: not specified	A	The partners perceive the attitude of the nurses as psychological pressure to continue home care even after they have reached their breaking point. Support is needed to enforce individual limits of care.
8	Ward-Griffin et al. (2000)	Canada	To examine the relationship between community nurses and family members caring for the elderly in the home – and the specific power relationships of nurses and family caregivers.	Focused in-depth interviews; thematic content analysis	*N* = 38 23 family caregiver-nurse dyads Age: Caregivers: 33–82 years (most over 60), Cared-for: 65–99 years Duration of marriage: not specified Duration of care: not specified	A, B, C	The relationship between the nurses and the caregiving partners does not work as a partnership (no “team”). The transition to working with a professional requires time and trust on the part of the caregiving partner and patience on the part of the nurses. Conflicting (role) expectations led to tensions between family caregivers and nurses.

Analysis of the content of the studies revealed the dynamics and diverse challenges involved in care triads. The results were structured according to the three research questions and are presented in Table 4, which links each question to the relevant thematic aspects identified in the data.

**Table 4:** Category system

Research question	Themes
1. How is collaboration within the care tried experienced by care recipients, caregiving partners, and nurses?	• Dynamic roles and relationships • Striving for independence by couples • Excessive demands on couples • Inadequate communication • Internal conflict faced by nurses (caught between feeling responsible for both partners)
2. How do those involved – care recipients, caregiving partners and nurses – view the support approaches adopted by nurses to the at-home care situations of couples?	• Reactive and insufficiently proactive • Emotional and instrumental support providing relief to couples • Psychological pressure to care for partner • Insufficient sensitivity towards same-sex partnerships
3. What are the facilitators of and barriers to collaboration within the care triad?	Facilitators: • Clear and open communication • Long-term relationship work, establishment of regular routines for care delivery • Proactive knowledge transfer • Processes adapted to the relationship dynamics Barriers: • Exclusion of couples from social contexts • Conflicts and tension due to unclear task distribution • Strain caused by nurses transferring tasks to the caregiving partner

### How is collaboration within the care tried experienced by care recipients, caregiving partners, and nurses?

The studies indicate consistently that the members of the care triad perceive and step into dynamic roles and relationships. The couples say that their primary aim is initially to maintain their independence from third-party support services for as long as possible despite limiting health conditions and to manage care without outside support. At times, they draw on help from neighbours and relatives [31, 32]. The nurses sometimes perceive a negative attitude on the part of the couples towards the support they provide in the home [33, 34]. When couples finally decide to involve professional home care services, all members of the triad enter a phase in which they strive for collegial cooperation [34]. In later phases, the couples and nurses perceive themselves in a relationship that has become closer [32, 35] and is characterised by mutual trust [31]. From the nurses’ perspective, however, providing care or securing its provision remains central and sharing decision-making is a lesser priority [32, 34]. Both informal caregivers and nurses complain about inadequate communication with each other as a problem that emerges over time [32, 34]. Dealing with home care services can overwhelm couples [32, 35].

Caregiving partners regard the support provided by nurses as a crucial element of quality care, particularly when it addresses both their practical needs and emotional well-being [34, 36]. Nurses report that they feel torn between the needs of direct-care recipients and those of informal caregivers. While care recipients require emotional support in addition to physical care, informal caregivers also describe themselves as “chronically tired” and “extremely tense” [34]. However, the caregiving partners do not want to see themselves cast in the role of patients and instead desire recognition of the skills they have acquired as they care for their spouses [34].

### How do those involved – care recipients, caregiving partners and nurses – view the support approaches adopted by nurses to the at-home care situations of couples?

Caregiving partners report that the routines of home care services often require them to adjust their own daily activities, which can lead to a loss of personal autonomy and time. This ongoing adaptation is experienced as burdensome, as everyday life becomes increasingly shaped by the demands of caregiving. In response, many seek to work with professionals to re-establish a sense of continuity and develop new, manageable structures for their day-to-day lives [36]. They experience nurses as overly reactive and would like to see nurses take their day-to-day needs into account more proactively [32, 34, 37]. Caregiving partners who are initially hesitant to involve professional care in their homes often come to recognize the value of such support over time – both for themselves and their partner [31, 38]. They experience not only physical relief but also emotional support and a growing sense of security as they navigate the caregiving situation [34]. An ongoing relationship with nurses marked by trust further reinforces the feeling of not being alone with the everyday challenges of caregiving [31, 34, 38].

In the study by Hudson et al. [35], nurses report that they are often confronted with emotionally charged and complex interactions when they work with the couples and that they attempt to approach these interactions sensitively and to stabilise the couple relationships. Many nurses expect caregiving partners to acquire caregiving competencies and actively teach them technical skills for managing routine care. While initial collaboration may be experienced as collegial, nurses often do not engage in shared decision-making but instead focus on ensuring that tasks are carried out. In this context, nurses position themselves in a (collegial) supervisory and controlling role. Nurses describe themselves as “resource persons” who provide information, emotional support, and access to additional resources to enable couples to maintain independence as long as possible [34].

However, the caregiving partners describe inadequate coordination between nurses and other professionals and services. They reported that this hinders cooperation in an atmosphere of mutual trust, as the expectations and contributions of all involved parties need to be clear to facilitate cooperation [32, 33]. Münch [37] reports in her study that caregiving partners feel that nurses pressure them to continue caring for their partners at home even when they have reached their breaking point. The homosexual caregiving partners interviewed in the study by Hash [33] criticise that nurses are not sufficiently sensitive towards their relationships [33]. They attribute nurses’ lack of sensitivity to the specific needs of same-sex couples to homophobia. However, the majority of the caregiving partners surveyed blame an impersonal healthcare system, which does little to reward relationship work, rather than nurses themselves for what they perceive as a lack of attention to their individual lives and care situations [33].

### What are the facilitators of and barriers to collaboration within the care triad?

Nurses and caregiving partners say that they see clear and open communication [31, 33, 38] and dealing with each other as partners as necessary for successful cooperation in the homes of clients [36]. Both couples and nurses value being able to address and resolve contradictory expectations (e.g. about roles) and confusion about who is responsible for what [31, 34] to avoid tension and conflicts. From the perspectives of all three parties, this requires a relationship characterised by mutual trust to be established that can give the couples stability in their everyday lives and a sense of security [34, 36, 38]. Couples desire continuity of personnel rather than “deployments” of different nurses so that shared routines can be developed and tasks can be entrusted to nurses with the confidence that has been built up over time [31, 35].

Caregiving partners consider the communication of instructions and knowledge by nurses decisive for strengthening their skills as caregivers in the home. From their perspective, this encompasses both the teaching of practical skills that enable the caregiving partner to provide appropriate assistance and the proactive communication of knowledge about the relevant health issue and the support which is available [34, 38]. They report that they need to work closely with nurses to sustain home-based care, uphold familiar routines as well as possible, and prepare for future developments [32]. Caregiving partners also report that nurses foster their self-care so that their physical, psychological and social needs can be met despite their responsibilities at home and they can continue their caregiving role [34]. It is advantageous for this if nurses are familiar with different kinds of couple relationships and can understand them and consider their special features [33].

Barriers for the collaboration of couples and nurses are also addressed in the studies. Accepting professional nursing care seems to be especially difficult when the couple is socially isolated and when the partners have few other social contacts [31]. Caregiving partners and nurses report that a lack of clarity about their respective responsibilities can lead to tension and conflicts between them [33, 34]. Ward-Griffin et al. [34] note that the partners can experience the tasks handed over to them by nurses as too complex and that this sometimes triggers fears and feelings of being overwhelmed. When the partners feel that they have been left to tackle tasks alone, they often react with “anger” towards nurses [36]. Other studies also show that dealing with home care services can be overwhelming for couples [32, 35].

## Discussion

This integrative review investigated the perceptions of nurses and older couples in the context of triadic care scenarios in home-based care. It analysed how those involved experience the care triad situation and perceive the approaches adopted by nurses to supporting couples in this care setting. It also looked at the factors that facilitate or hinder cooperation in care triads. The results confirm that building and maintaining relationships is central for the success of care triads [22]. The importance of relationship-building in care triads is also underscored by a study of quality in home-based care involving care recipients, informal carers, and nurses [39] that shows a preference for cooperation marked by trust over purely task-oriented forms of interaction. Multiple studies included in this review show that older couples are often initially hesitant to involve home care services because of their desire to preserve privacy in their homes and to maintain familiar routines and roles that have evolved over many years (e.g [32, 35]). The results of a study of older couples in Sweden living in long-term caregiving relationships due to physical disabilities indicate that nurses and other professionals providing services can access the lifeworlds of couples in caregiving relationships effectively by supporting the continuation of the familiar routines of the couples [40]. Soodeen et al. [41] also emphasise that the preservation of familiar everyday routines is a key factor for the acceptance of home care services. Our results also show – in line with Nolan’s Senses Framework [23] – the importance of care that is characterised by a sense of continuity. Regular support from a personal nurse who shows esteem for the couple’s shared life narrative and supports their shared routines creates this sense of continuity. A nurse’s support can bolster the psychological stability of the dyad by giving couples a reassuring feeling that they do not have to face the challenges of the care situation alone. Staffing continuity in home-based care is also associated with positive effects on the health and well-being of care recipients: fewer falls, a higher probability of functional improvement or stabilisation, and fewer depressive symptoms [42]. Relationship work poses special challenges in dementia care, as the nature of interactions, mutual understanding and communication changes in this context. Due to their cognitive impairments, people with dementia require specific forms of communication and relationship-building that are more complex than the interactions involved in routine nursing care. This demands specific communication skills, high levels of emotional sensitivity, and a strongly empathic presence from nurses. Providing a supportive and stable care environment by taking targeted action both to involve spouses and to provide them with relief also takes on particular importance in these settings [11]. The studies included in the review show that support from nurses gives couples a sense of security, a point which is also emphasised in Nolan’s model of relationship-centred care [17, 22]. Older people in need of care who have this sense of security feel well looked after both physically and emotionally [34]. For caregiving partners, this means receiving recognition in their caregiver role, learning from nurses, and having the opportunity to take a step back when necessary [32, 36]. As roles, relationships and needs within care triads usually evolve dynamically rather than remaining static, relationship-centred care is a process and phase-specific interventions are important, as Nolan et al. [43] observe. To be able to step back from the care situation temporarily, informal carers need to have confidence in the quality of care provided by nurses [7]. Emotional support from nurses and relief for the caregiving partner play a significant role in stabilising the triadic care relationship [34, 36] and maintaining a balanced and sustainable nursing care dynamic [44]. Caregiving partners of advanced age and with health limitations of their own are especially likely to be strained by caregiving responsibilities and to jeopardise their own health when they care for a partner. Studies included in the literature review such as that by Münch [37] – but also a further study by Woodman et al. [45] – show that informal carers feel overwhelmed and come under pressure from nurses who appeal to their sense of duty in an effort to ensure that care at home is sustained for as long as possible. When too many tasks are delegated to caregiving partners and responsibilities are not clearly distributed, relationships between caregiving partners and nurses can become tense [33, 34]. Most of the studies included in this review emphasise that nurses working with older couples are often internally conflicted: their desire to secure the best possible care for the care recipients can prompt them to involve caregiving partners more closely, but they also recognise the vulnerability of caregiving partners and their need for relief and want to foster their well-being. Nurses often feel a sense of responsibility for maintaining stability in the triad, especially when caregiving partners appear exhausted or emotionally burdened [34, 35]. For nurses, as the study by Wälivaara et al. [46] on relationships in home care shows, this means setting boundaries when the relationship becomes too personal and the distance necessary for a professional relationship is in danger of being lost.

The results of our study also show that the possibilities for shaping the individual relationships between clients and nurses are influenced by the organisational and working conditions of home care services. Haex et al. [39] emphasise that the capability of nurses to contribute to a stable dynamic characterized by trust within the triad is limited by structural constraints such as staff shortages and fragmented teams. As Eurofound [47] notes, almost half of the nursing staff working in long-term care in the UK leave their jobs within the first year of employment, and an OECD report [48] shows that the time since the Covid pandemic has been characterised by deteriorating working conditions and rising staff turnover. When changes of nursing staff are frequent, it is difficult for relationships marked by warm and consistent communication between nurses and couples to become established. Studies have confirmed that staffing changes can lead to care recipients and their caregiving partners feeling anxious and uncertain [12, 41]. Nurses themselves emphasise the importance of stable relationships marked by trust for fostering smooth cooperation (see also: 49, 50). Remuneration systems that fail to recognise relationship work as part of the process of delivering nursing care clash with the ethical and professional self-image of nurses who desire to provide holistic and not purely task-oriented care [51, 52].

Our results highlight that the focus of triadic nursing care in the home cannot be placed purely on accomplishing tasks involved in care delivery but must also be on building sustainable relationships and creating a sense of security by keeping personnel consistent, supporting familiar routines and laying down foundations for successful cooperation.

## Strengths and limitations of the review

The data basis for answering the research questions is relatively small, with eight studies included, and this limits the significance of the results. This is especially true in the context of the expanding sexual and gender diversity of older people [53]. Only one of the eight studies looked at the experiences of same-sex older couples working with nurses. In general, the results of this review point to a research gap in the area of qualitative studies on care triads involving nurses and older couples. A further limitation of this review lies in the search strategy: studies that did not explicitly state a focus on (predominantly) older spousal caregiving couples could not be identified and included. As a result, relevant studies such as Haex et al. [39] were not retrieved through our search in PubMed (via MEDLINE) and CINAHL, even though they align with our inclusion criteria. Although all of the included studies conceptually refer to the care situation as a triad involving the caregiving partner, the care recipient and nurses, the perspective of nurses themselves is surprisingly underexplored. Only two of the selected articles [34, 35] explicitly examine the views of nurses. This noticeable imbalance points to a significant research gap: while the topic of couple-based caregiving in the home setting has received limited attention overall, the professional perspective of nurses remains particularly underrepresented. Given the central role that nurses play in supporting care relationships at home, future research should more thoroughly incorporate their views. Future research should also go beyond describing problems and seek to identify effective strategies for fostering collaboration in care triads in home-based nursing care.

## Conclusions

The present review illustrates the central role of the formation and maintenance of relationships in care triads in the home composed of a person in need of long-term care, a caregiving partner and a nurse. Building strong relationships is a key factor in the successful integration of professional nursing care. As older couples are often reluctant to integrate home care services into their established routines, nurses recognising and considering these existing routines is a key prerequisite for supporting couples effectively. In summary, the perspective of nurses on relationship-building, emotional burden and structural limitations within triadic care constellations with older couples needs further investigation.

The results of the review also show that the delivery of care by consistent care teams or personal nurses strengthens the trust of both the caregiving partner and the care recipient. This not only improves the quality of the nursing relationship but also gives informal caregivers some temporary respite from their caregiving roles and a sense of security. As roles, relationships and needs within the care triad evolve dynamically, phase-specific interventions and relationship-centred care approaches have special importance for meeting these changing needs.

Because caregiving partners are often under considerable strain and of advanced age, the support provided by nurses should always include targeted action to relieve their caregiver burden. Organisational and structural conditions (including a lack of time and high workloads) make it difficult for nurses to practically implement relationship-centred care despite wide recognition of the value of continuity in relationships. Shortages of skilled staff and cost pressures also present barriers to the delivery of care models centring relationships. Nurses therefore face the complex task of addressing the individual needs of clients despite unfavourable organisational conditions.

If the goal of ageing in place is to be supported successfully and become reality, it is essential to consider the specific needs of nurses, caregiving partners and care receivers and to modify the prevalent conditions in ways that foster relationship work in care triads effectively.

## Electronic supplementary material

Below is the link to the electronic supplementary material.

Supplementary Material 1

## Data Availability

No datasets were generated or analysed during the current study.

## Footnotes

1 In this context, the term *nurses* refers to professionals providing nursing care in the home; the authors acknowledge that roles and qualifications may vary across countries and care systems.

2 First search: 25 August 2022. Follow-up searches were carried out on 28 June 2024.

3 In the studies of Münch (2020) and Lindgren (1993), the involvement of professional nurses is not clearly specified, but both studies relate to home care situations that commonly involve professional nurses. Both studies were included for their relevance to caregiving couples, but not analysed for statements on nurse-led triadic collaboration.

## Abbreviations

CINAHL: Cumulative Index to Nursing and Allied Health Literature
MEDLINE: Medical Literature Analysis and Retrieval System Online
PICo: Population, Phenomenon of Interest and Context
PRISMA: Preferred Reporting Items for Systematic Reviews and Meta-Analyses
WHO: World Health Organization
