# Supplementary material for: Triadic relationships in home care nursing: an integrative review of the views and experiences of older couples and nurses
Source: BMC Nurs. 2025 Jun 27;24:671. doi: 10.1186/s12912-025-03378-1 (PMC12203712; doi:10.1186/s12912-025-03378-1)
Supplement: Supplementary file 1 — Supplementary Material 1 [file 12912_2025_3378_MOESM1_ESM.pdf]

Additional File Search Strategy

PubMed

((((((((((((Home Care Services[MeSH Terms]) OR ("Home Care Services"[Title/Abstract])) OR ("Domiciliary car*"[Title/Abstract])) OR ("Home Health Nurs*"[Title/Abstract])) OR ("Home Health Car*"[Title/Abstract])) OR ("home healthcare"[Title/Abstract])) OR ("Home Nurs*"[Title/Abstract])) OR ("home car*"[Title/Abstract])) OR ("care at home"[Title/Abstract])) OR ("Family car*"[Title/Abstract])) OR ("relative*"[Title/Abstract]) AND ((english[Filter] OR german[Filter]) AND (1992:2022[pdat]))) AND (((((((("Marriage"[MeSH Terms]) OR (spouses[MeSH Terms])) OR ("spous*"[Title/Abstract])) OR ("couple*"[Title/Abstract])) OR ("partner*"[Title/Abstract])) OR (wife[Title/Abstract])) OR ("wive*"[Title/Abstract])) OR ("husband*"[Title/Abstract]) AND ((english[Filter] OR german[Filter]) AND (1992:2022[pdat])))) AND (((("Aged"[MeSH Terms]) OR ("Aged"[Title/Abstract])) OR ("old person*"[Title/Abstract])) OR ("elder*"[Title/Abstract]) AND ((english[Filter] OR german[Filter]) AND (1992:2022[pdat])))) AND ((((((Nursing Care[MeSH Terms]) OR (Nursing[MeSH Terms])) OR (family nursing[MeSH Terms])) OR ("community nurs*"[Title/Abstract])) OR ("community health nursing"[MeSH Terms])) OR ("public health nursing"[MeSH Terms]) AND ((english[Filter] OR german[Filter]) AND (1992:2022[pdat])))

Filters: English, German

CINAHL

(TI “home care services” OR AB “home care services” OR TI “domiciliary car*” OR AB “domiciliary car*” OR TI “home health nurs*” OR AB “home health nurs*” OR TI “home health car*” OR AB “home health car*” OR MH "Home Health Care" OR TI “home healthcare” OR AB “home healthcare” OR TI “home nurs*” OR AB “home nurs*” OR TI “home car*” OR AB “home car*” OR TI “care at home” OR AB “care at home” OR TI “Family car*” OR AB “Family car*”) AND (TI marriage OR AB marriage OR TI spous* OR AB spous* OR TI couple* OR AB couple* OR TI partner OR AB partner OR TI wife OR AB wife OR TI wive* OR AB wive* OR TI husband OR AB husband OR TI relative* OR AB relative*) AND (TI aged OR AB aged OR MH "Aged+" OR TI “old* person*” OR AB “old* person*” OR TI elder* OR AB elder*) AND (TI “nursing care” OR AB “nursing care” OR MH "Nursing Care" OR TI nursing OR AB nursing OR TI “family nursing” OR AB “family nursing” OR MH "Family Nursing" OR TI “community nurs*” OR AB “community nurs*” OR TI “community health nursing” OR AB “community health nursing” OR (MH "Community Health Nursing") OR TI “public health nursing” OR AB “public health nursing”)
